# Global omics study of *Tetraselmis chuii* reveals time-related metabolic adaptations upon oxidative stress

**DOI:** 10.1007/s00253-023-12936-z

**Published:** 2024-01-16

**Authors:** Aikaterini Koletti, Dimitrios Skliros, Chrysanthi Kalloniati, Sofia Marka, Maria-Eleftheria Zografaki, Carlos Infante, Lalia Mantecón, Emmanouil Flemetakis

**Affiliations:** 1 Department of Biotechnology, School of Applied Biology and Biotechnology, Agricultural University of Athens, 11855 Athens, Greece; 2 Department of Marine Sciences, University of the Aegean, University Hill 81100, Mytilene, Greece; 3 Fitoplancton Marino, S.L., Dársena Comercial S/N (Muelle Pesquero), 11500 El Puerto de Santa María (Cádiz), Spain

**Keywords:** Microalgae, *T. chuii*, Oxidative stress, H_2_O_2_, Multi-omics

## Abstract

**Abstract:**

Microalgae species encounter oxidative stress in their natural environments, prompting the development of species-specific adaptation mechanisms. Understanding these mechanisms can offer valuable insights for biotechnological applications in microalgal metabolic manipulation. In this study, we investigated the response of *Tetraselmis chuii*, an industrially important microalga, to H_2_O_2_-induced oxidative stress. Exposure to 0.5-mM H_2_O_2_ resulted in reduced cell viability, and higher concentrations led to a drastic decline. After 1 h of exposure to H_2_O_2_, photosynthetic capacity (Qy) was negatively impacted, and this reduction intensified after 6 h of continuous stress. Global multi-omics analysis revealed that *T. chuii* rapidly responded to H_2_O_2_-induced oxidative stress within the first hour, causing significant changes in both transcriptomic and metabolomic profiles. Among the cellular functions negatively affected were carbon and energy flow, with photosynthesis-related *PSBQ* having a 2.4-fold downregulation, pyruvate kinase decreased by 1.5-fold, and urea content reduced by threefold. Prolonged exposure to H_2_O_2_ incurred a high energy cost, leading to unsuccessful attempts to enhance carbon metabolism, as depicted, for example, by the upregulation of photosystems-related *PETC* and *PETJ* by more than twofold. These findings indicate that *T. chuii* quickly responds to oxidative stress, but extended exposure can have detrimental effects on its cellular functions.

**Key points:**

• *0.5-mM H*_*2*_*O*_*2*_*–induced oxidative stress strongly affects T. chuii*

• *Distinct short- and long-term adaptation mechanisms are induced*

• *Major metabolic adaptations occur within the first hour of exposure*

**Supplementary Information:**

The online version contains supplementary material available at 10.1007/s00253-023-12936-z.

## Introduction

Microalgae are unicellular organisms that inhabit all aquatic realms on Earth (Park et al. 2022). As they have adapted to different environments, microalgae can tolerate extreme conditions by utilizing their metabolic plasticity including biosynthesis of a wide range of different metabolites (Park et al. 2022). This attribute can lead to their potential application as feedstocks, for producing nutraceuticals, pharmaceuticals, and biofuels (Bhalamurugan et al. 2018). The induction of oxidative stress responses is a common metabolic adaptation strategy for all microorganisms, controlled either by intracellular processes or by environmental stimuli, as reactive oxygen species (ROS) are well-known molecules accumulating under stress conditions (Cirulis et al. 2013). ROS, like hydrogen peroxide (H_2_O_2_), act via the oxidation of cellular nucleic acids, proteins, and fatty acids and lead to imbalance of cell life if not properly neutralized (Almeida et al. 2017). Due to their particular and diverse lifestyles and habitats, green microalgal species have developed a substantial repertoire of antioxidant mechanisms, which scavenge excess oxidants or free radicals to prevent the deleterious effects of ROS (Barone et al. 2021). This repertoire includes anti-ROS enzymes such as catalase, superoxide dismutase, and glutathione peroxidase as well as various antioxidant compounds, like astaxanthin and lutein (Mishra and Jha 2011; Lu et al. 2021).

Understanding how oxidative stress reprograms metabolic machinery of microalgal cells is of particular interest for industry, as long-term exposure to ROS can lead to the collapse of a culture (Lu et al. 2021). Additionally, within the concept of metabolic manipulation, induced oxidative stress represents a promising strategy in order to enhance the production and accumulation of desired high-value biomolecules with potent antioxidant activity. Several stressors can be utilized for the induction of oxidative stress, including hydrogen peroxide (Qiao et al. 2021). In this scenario, it has been reported that the green microalga *Chlamydomonas reinhardtii* responded by inducing global alterations in its cellular and metabolic machinery within 6 h upon H_2_O_2_-induced oxidative stress, a regulatory process involving the *Cr*SBP selenium-binding protein (Koletti et al. 2022). Similarly, H_2_O_2_-induced oxidative stress resulted in the differential regulation of several transcripts in *C. reinhardtii* (Blaby et al. 2015). During the past years, it has been shown for different microalgal species that H_2_O_2_ effectively induces stress and initiates metabolic alterations, yet in a clearly species-specific manner (Barone et al. 2021). Environmental studies proved that green microalgae, like *Chlorella* sp., degrade H_2_O_2_ much faster than various cyanobacteria species (Weenink et al. 2021). Furthermore, inclusion of *Tetraselmis chuii* on *Litopenaeus vannamei* post larvae feed had a positive effect on oxidative stress status, survival, and resistance to salinity stress, constituting a promising option for the improvement of aquaculture production of this crustacean (Rahman et al. 2017).

In this study, we have employed a multi-omics approach to gain insights into the molecular and biochemical adaptations of *T. chuii* under H_2_O_2_-induced oxidative stress. *T. chuii* is a flagellated microalgal species belonging to *Chlorophyta* and is commonly utilized as feedstock due to its rich composition of antioxidants (Rahman et al. 2017). Despite the increasing interest in *T. chuii* in recent years, its specific molecular and metabolic adaptation strategies against abiotic stresses remain elusive. Our results demonstrate that *T. chuii* can initiate both rapid and effective responses as an adaptation to H_2_O_2_-induced stress. However, prolonged exposure to H_2_O_2_ leads to metabolic dysregulation.

## Materials and methods

### Strains and growth conditions, H_2_O_2_-induced oxidative stress

*T. chuii* CCFM-03 (deposited and available at the Culture Collection of the company Fitoplancton Marino, S.L., Cádiz, Spain) was grown on f/2 culture medium (Guillard and Ryther 1962). Liquid cultures (1-L final volume) were grown under constant illumination (30 μmol m^−2^ s^−1^), with constant supplementation of filtered air and stirring at 25 °C. Stock cultures at exponential growth were used for flask inoculation and initial cell density of the cultures was 1 × 10^6^ cells mL^−1^. Based on a pilot experiment (data not shown), it was decided to use the concentrations of 0-mM (control), 0.5-mM, 0.7-mM, 1-mM, and 1.5-mM H_2_O_2_ including three biological replicates. When cultures reached exponential phase (2 × 10^6^ cells mL^−1^), the corresponding amount of freshly aliquoted H_2_O_2_ (AppliChem, Gatersleben, Germany) was added to each culture for the respective concentration. For the identification of cell density and culture viability assay, samples were collected right before the addition of H_2_O_2_ (0 h), 6, and 12 h later (6 h, 12 h). At time points 0, 1, and 6 h after the addition of H_2_O_2_, biomass was collected and was instantly lyophilized.

### Cell growth kinetics

Determination of cell density and viability (in cells mL^−1^) was performed with a Neubauer chamber (HBG, Giessen, Germany) and a fluorescent microscope Zeiss AX10 (Carl Zeiss, Oberkochen, Germany) with FITC filter (excitation 450–490 nm, emission 515–565 nm). More specifically, for the determination of cell viability, fluorescein diacetate (Sigma-Aldrich, Steinheim, Germany) was utilized, with final concentration 5 μg mL^−1^ and incubation for 5 min at room temperature alongside with 1 μL Lugol (Sigma-Aldrich, Steinheim, Germany). The cell number was calculated following the manufacturer’s instructions and expressed as 10^6^ cells mL^−1^.

### Chlorophyll fluorescence measurements and photometric assays

Photosystem II (PSII) maximum quantum yield (Qy) was determined by measuring the chlorophyll fluorescence in a portable pulse amplitude modulation (PAM) fluorimeter (AquaPen AP-100, Photon Systems Instruments, Drásov, Czech Republic) according to the user’s manual. Cultures were adapted to dark conditions for 10 min prior to measurement.

Freeze-dried microalgal biomass and the Infinite 200 PRO plate reader (Tecan, Männedorf, Switzerland) were utilized for the photometric assays. Total lipid content was determined with the sulfo-phospho-vanillin reaction (SPV) method, as described by Mishra et al. (2014). The total polysaccharide content was determined through the phenol–sulfuric acid method, following the procedure outlined by DuBois et al. (1956). To estimate the content of total carotenoids and chlorophylls a and b, pigment extraction was performed using 80% acetone, as per the protocol by Lichtenthaler and Wellburn (1983). For assessing the antioxidant capacity, the ferric reducing antioxidant power (FRAP) assay was conducted using 100% methanol as the solvent, in accordance with the method of Re et al. (1999). The same solvent was used to estimate the phenolic content using the Folin-Ciocalteu method (Jan et al. 2013).

### RNAseq transcriptomic analysis

At each timepoint and for each culture, total RNA was isolated using NucleoZOL (Macherey–Nagel, Dueren, Germany) following the manufacturer’s protocol. A DNAse reaction (Turbo DNase, Invitrogen, Carlsbad, CA, USA) followed, and RNA was quantified by spectrophotometry (NanoDrop ND-1000, Thermo Fischer Scientific, Waltham, MA, USA) and agarose gel electrophoresis. Illumina TruSeq RNA Sample Preparation Kit v2 (Illumina, San Diego, CA, USA) was used for the preparation of RNA libraries, according to the manufacturer’s protocol. For sequencing, a NextSeq 500 unit (Illumina, San Diego, CA, USA), 150 single-end run was used. Trimming ligands, adapters, and quality filtering followed up, and clean reads were mapped under Geneious platform (R10 version; Biomatters Ltd., Auckland, New Zealand (Kearse et al. 2012)) to the *T. chuii* strain PLY429 transcriptome, available in the iMicrobe database (https://www.imicrobe.us/). Quantification of gene expression levels was estimated by reads per kilobase of transcript per million fragments mapped method (RPKM). Deseq2 software was utilized for differential expression and statistical analysis of mapped reads (Love et al. 2014).

BlastKOALA was used to perform KO (KEGG Orthology) assignments and characterize DEGs (differentially expressed genes) (Kanehisa et al. 2016). Annotated KOs and gene IDs were complemented with results from eggnog-mapper v2 annotation (Cantalapiedra et al. 2021). The associated KEGG pathways were retrieved using KEGG mapper (Kanehisa et al. 2022), and enrichment analysis for KEGG pathways was done with g:Profiler (Kolberg et al. 2023). Functional characterization of DEGs for GO terms was done with PANTHER classification system (Mi et al. 2021).

### RT-qPCR-based transcript analysis

To confirm the observed transcriptome profiles, real-time RT-qPCR analysis was performed. Five genes involved in several metabolic pathways or coding for key proteins were selected, and their relative expression patterns are presented in Supplemental Fig. S1.

Total RNA was isolated from a new batch of cultures, as already described, with three biological replicates. For cDNA synthesis, Super-Script II (Invitrogen, Carlsbad, CA, USA) and oligo (dT) primers were used, with 5 ng of RNA as a starting material. For the RT-qPCR analysis, StepOnePlus Real-Time PCR System (Applied Biosystems, Foster City, CA, USA) was employed, with SYBR Green with ROX PCR Master Mix (Applied Biosystems, Foster City, CA, USA) and gene-specific primers. Primers were designed with Primer Express software (Applied Biosystems, Foster City, CA, USA) (Supplemental Table S1). Primer specificity and formation of primer dimers were monitored by dissociation curves. PCR cycling started at 95 °C for 10 min, followed by 40 cycles of 95 °C for 15 s and 60 °C for 1 min, with reaction volume set at 10 μL. For the calculation of the relative change in transcript levels, the 2^–ΔCt^ method based on the cycle threshold values was used, normalized to the internal control genes, *Tc*cdkA and *Tc*UBCE (Torres et al. 2021). Amplification efficiency was calculated by employing the linear regression method on the log (fluorescence) per cycle number data, using LinRegPCR software (Ramakers et al. 2003).

### Gas chromatography-mass spectrometry (GC–MS)–based untargeted metabolomic analysis

Microalgal cells were harvested by centrifugation (4 min, 2,800 rpm, 4 °C), resuspended in 1-mL f/2, and the collected biomass was lyophilized and stored at − 20 °C. Metabolites of each lyophilized sample were extracted from 50 mg as previously described (Patelou et al. 2020). For each of the three independent biological replicates, two metabolite profiles were obtained (*n* = 6).

For GC–MS analysis, the methanol extraction method was used. Liquid nitrogen was employed to pulverize 50 mg of lyophilized microalgal biomass, which was further grounded with 395 μL of ice-cold methanol and 5 μL of 1 mg/mL ribitol. Incubation at 70 °C for 15 min under continuous shaking followed, and then, 200 μL chloroform was added to the samples, followed by incubation at 37 °C for 5 min under continuous shaking. Thereafter, 400 μL of ddH_2_O was added, and samples were vigorously vortexed and centrifuged at 13,000 rpm for 5 min at room temperature. The aqueous phase containing the polar metabolite fraction was evaporated under nitrogen conditions to avoid oxidation of metabolites from atmospheric oxygen. For derivatization, dried samples were resuspended in methoxyamine-HCl (20 mg mL^−1^ pyridine) and incubated for 90 min at 30 °C with vigorous shaking. After centrifuging at 13,000 rpm for 30 s, 75 μL of *N*-methyl-*N*-(trimethylsilyl)-trifluoroacetamide (MSTFA, Sigma-Aldrich, Steinheim, Germany) was added. A final incubation for 30 min at 37 °C followed, with centrifugation at 13,000 rpm for 2 min at room temperature. GC–MS analysis was carried out on a gas chromatographer (Agilent 7890A, Santa Clara, CA, USA) coupled to a mass spectrometer (Agilent 5973C, Santa Clara, CA, USA). For the separation of the metabolite fragments, the capillary column HP-5MS (30 m, 0.25 mm ID, film thickness 0.25 μm, Agilent) was used. The carrier gas was helium, with a flow rate of 1 mL min^−1^. A FAME mix (Sigma-Aldrich, Steinheim, Germany) was injected separately for the determination of retention indexes (RIs). Pulsed split mode of injection was used, with the injection volume at 1 μL. The GC inlet temperature was 230 °C and total run time was 61 min. The oven temperature program was as follows: initial temperature 80 °C for 3 min, followed by increase with rate 5 °C/min to 320 °C and held for 10 min. The MS source was held at 250 °C and the quadruple at 150 °C, scanned from m/z 83 to 500. For the extraction of the compounds analyzed by GC–MS, AMDIS (Automated Mass Spectral Deconvolution & Identification System) software, provided by the NIST (Gaithersburg, MD, USA), was used. The Fiehnlab compound libraries (Fiehn et al. 2011) were used for the identification of each component, according to its mass spectrum and retention time.

### Statistical analysis

To evaluate statistical significance, the IBM SPSS Statistics 23 (IBM, Armonk, NY, USA) was used to accomplish analysis of variance (ANOVA), followed by Tukey’s HSD (honestly significant difference) multiple comparison test at a 95% level of significance (*p* < 0.05). Partial least squares discriminant analysis (PLS-DA) and principal component analysis (PCA) were performed using the Multibase 2015 add-in for Excel (Microsoft, Redmond, WA, USA). Graphical representations were created with software SigmaPlot 12.0 (Systat software; SSPS Inc, Chicago, IL, USA) and pie charts with Excel. Pathway map representation and summarizing figure were created in BioRender.com (YE2656HHTO, GO264QH0SI; https://www.biorender.com/). In regard to RNA sequencing, thresholds of the significant differential expression were set as adjusted *p* < 0.05 and differential expression absolute confidence > 6.

## Results

### H_2_O_2_-induced oxidative stress effect on T. chuii cell viability

**Fig. 1:**
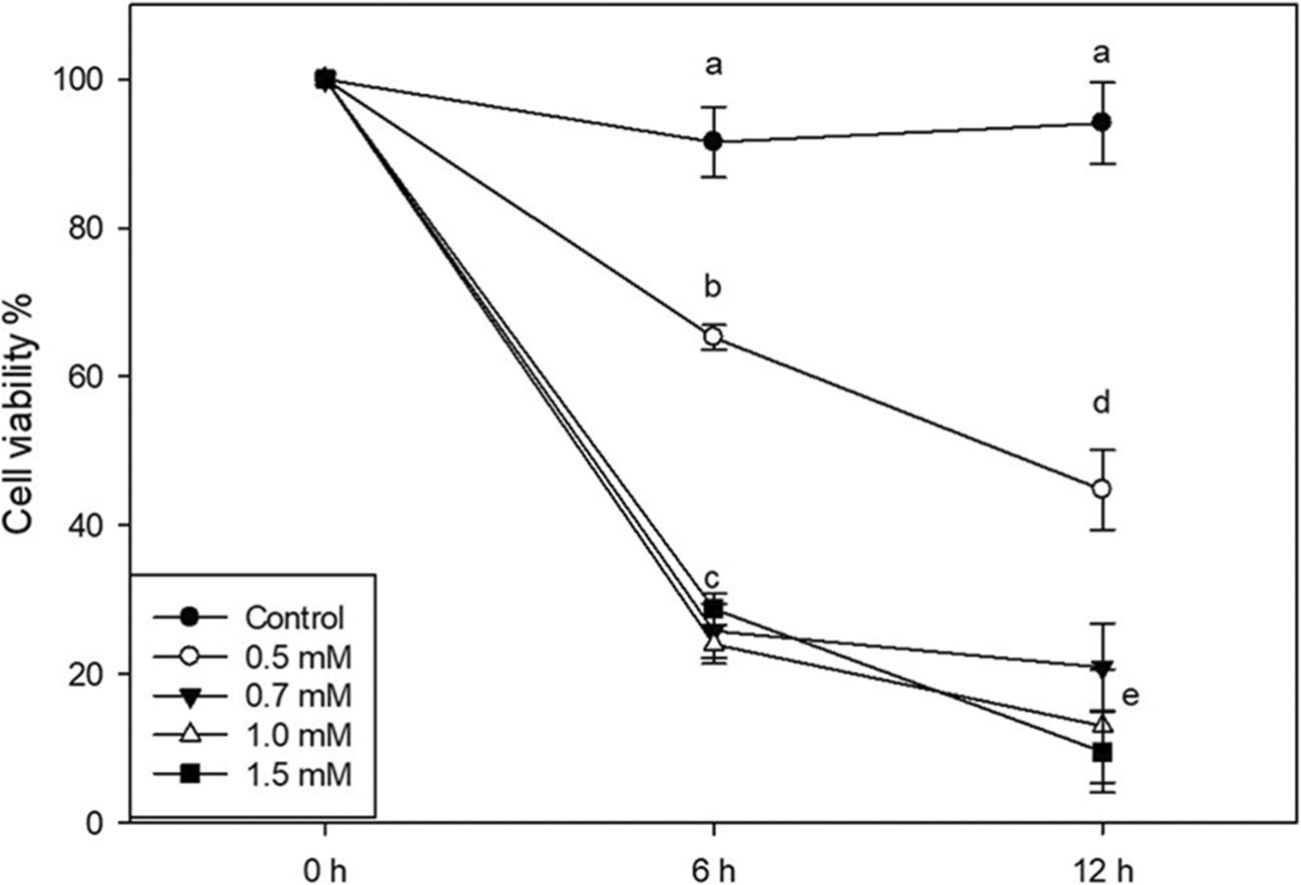
Cell viability of *T. chuii* exposed to different H_2_O_2_ concentrations. *T. chuii* cells were grown on f/2 medium in a bioreactor at 25 °C with constant supplementation of filtered air and illumination. When cultures reached exponential phase (timepoint 0 h starting point), H_2_O_2_ was added at final concentrations of 0 (control), 0.5, 0.7, 1, and 1.5 mM. Cell viability measurements were performed after 6 h and 12 h. All cultures were grown starting from the same inoculum density. Experiments were performed on batch cultures and viability was monitored with fluorescence microscopy. Data are shown as average ± SE of three biological repeats (*n* = 3). Statistically significant differences are indicated by different letters (Tukey’s HSD, *p* < 0.05)

To elucidate the effects of H_2_O_2_-induced oxidative stress on cell growth of *T. chuii*, five different concentrations of H_2_O_2_ were assessed. Exposure to 0.5 mM H_2_O_2_ for 6 h resulted in a statistically significant decrease in cell viability compared to the control. However, the viability remained higher than that observed at concentrations of 0.7-mM, 1-mM, and 1.5-mM of H_2_O_2_ (Fig. 1). Prolonged exposure to H_2_O_2_ for 12 h led to a further statistically significant decrease in cell viability. Cultures exposed to 0.5-mM H_2_O_2_ reached only 45% of the viability observed in control cultures. Moreover, viability for cultures exposed to higher H_2_O_2_ concentrations was lower than 20% (Fig. 1). Based on these findings, the application of 0.5-mM H_2_O_2_ for up to 6 h was considered an optimal condition to induce oxidative stress for further study of *T. chuii* response.

As an indicator of photosynthesis capacity, the maximal photosystem II quantum yield (Fv/Fm) was used to measure the conversion efficiency of light energy in the PSII reaction center. Exposure to 0.5-mM H_2_O_2_ for 1 h led to a statistically significant decrease in the Fv/Fm ratio, from 0.67 in the control cultures to 0.51 in the H_2_O_2_-stressed cultures. Prolonged exposure for 6 h caused a further decrease to 0.37, as shown in Table 1. A similar pattern of great decrease, as duration of exposure increases, was observed for the total carotenoids content of the cells. Also, chlorophyll b was decreased from 6.57 mg/g dry biomass in the control cultures to 4.97 mg/g dry biomass after 6 h of exposure to 0.5-mM H_2_O_2_. These results indicated a significant decrease in photosynthesis after 6 h under these mild oxidative stress conditions. An indication of the changes that occurred in the cells’ antioxidant potential was the decrease in FRAP values after 6-h exposure. In addition, a similar decline was observed in the important biochemical compositions of lipids and polysaccharides. Specifically, total lipid content of the cells remained stable for the first hour, at the levels of 14% of dry weight, while it dropped to 10.8% after 6 h. Total polysaccharides were 35.85% of dry weight in control cultures and were decreased to 28.51% 6 h later.

**Table 1:** Values (± SE) of photometric and colorimetric assays after exposure to H_2_O_2_ for 0 h, 1 h, and 6 h^1^

	FRAP (mg ascorbic acid/g dw)		TPC (mg gallic acid/g dw)		TL (% of dw)		TS (% of dw)		TC (mg/g dw)		Chl a (mg/g dw)		Chl b (mg/g dw)		Fv/Fm	
Treatment	AVG	SE	AVG	SE	AVG	SE	AVG	SE	AVG	SE	AVG	SE	AVG	SE	AVG	SE
0 h	24.00^a^	0.86	3.59	0.09	15.72^a^	0.21	35.85^a^	1.41	5.67^a^	0.1	8.86	0.27	6.57^a^	0.11	0.67^a^	0.02
1 h	24.05^a^	0.74	3.33	0.07	13.87^a^	0.4	30.27^a^	0.44	5.09^b^	0.07	7.53	0.21	6.01^a^	0.2	0.51^b^	0.01
6 h	14.55^b^	0.85	2.06	0.06	10.79^b^	0.49	28.51^b^	0.74	2.55^c^	0.1	5.58	0.23	4.97^b^	0.2	0.37^c^	0.04

^*1*^*FRAP* ferric-reducing antioxidant power; *TPC* total phenolic content; *TL* total lipids; *TS* total polysaccharides; *TC* total carotenoids; *Chl a* chlorophyll a; *Chl b* chlorophyll b; *dw* dry weight. For each treatment, ratio was calculated from the average of two biological and two technical repeats (*n* = 4). Different superscript letters indicate statistically significant difference among treatments

### Global comparative transcriptomic responses upon H_2_O_2_-induced oxidative stress

To gain an insight into the molecular adaptation mechanisms that govern the oxidative stress responses of *T. chuii*, RNAseq-based global transcriptomic profiling was utilized. For this purpose, high-quality total RNA samples were isolated from *T. chuii* cultures exposed for 1 and 6 h to 0.5-mM of H_2_O_2_. Cultures without the addition of the stressor were used as control. For each of the constructed libraries at least 12 million clean reads were generated with an average of 41% being successfully mapped to the reference transcriptome. Mapping resulted in the quantification of over 22,500 unique coding sequences (CDSs) in total (Supplemental Table S2). Out of these, 1761 CDSs were highlighted as differentially expressed genes (DEGs) between different treatments. Further analysis revealed that 501 DEGs were annotated with KOs (KEGG Orthology identifiers) and mapped to pathways (Supplemental Fig. S2). In addition, the functional annotation results, based on Gene Ontology (GOs), indicated that 41.4% of the DEGs were unclassified, while the remaining were involved in several molecular functions, with binding and catalytic activity being the most prevalent (Supplemental Fig. S3). To clarify the global response of *T. chuii* to the oxidative stress, enrichment analysis for GO annotation in the three categories biological process, cellular component, and molecular function as well as for KEGG pathways was obtained (Supplemental Fig. S4, S5). For 1-h exposure, in the KEGG pathways, ribosome was upregulated, while metabolic pathways and biosynthesis of secondary metabolites were highly downregulated. In the GO biological process category, protein metabolic process was enriched, and generation of precursor metabolites and energy was greatly decreased. In the GO molecular function category, structural molecule activity was the mostly upregulated. In the GO cellular component category, cytosol and ribonucleoprotein complex were highly enriched while plastid and chloroplast were severely downregulated. For 6-h exposure to oxidative stress, in the KEGG pathways, ribosome was enriched, and metabolic pathways were decreased. In the GO biological process category, protein metabolic process was highly upregulated, and photosynthesis was decreased. In the GO molecular function category, structural molecule activity was enriched. In the GO cellular component category, protein-containing complex was upregulated, while plastid and chloroplast were downregulated.

**Fig. 2:**
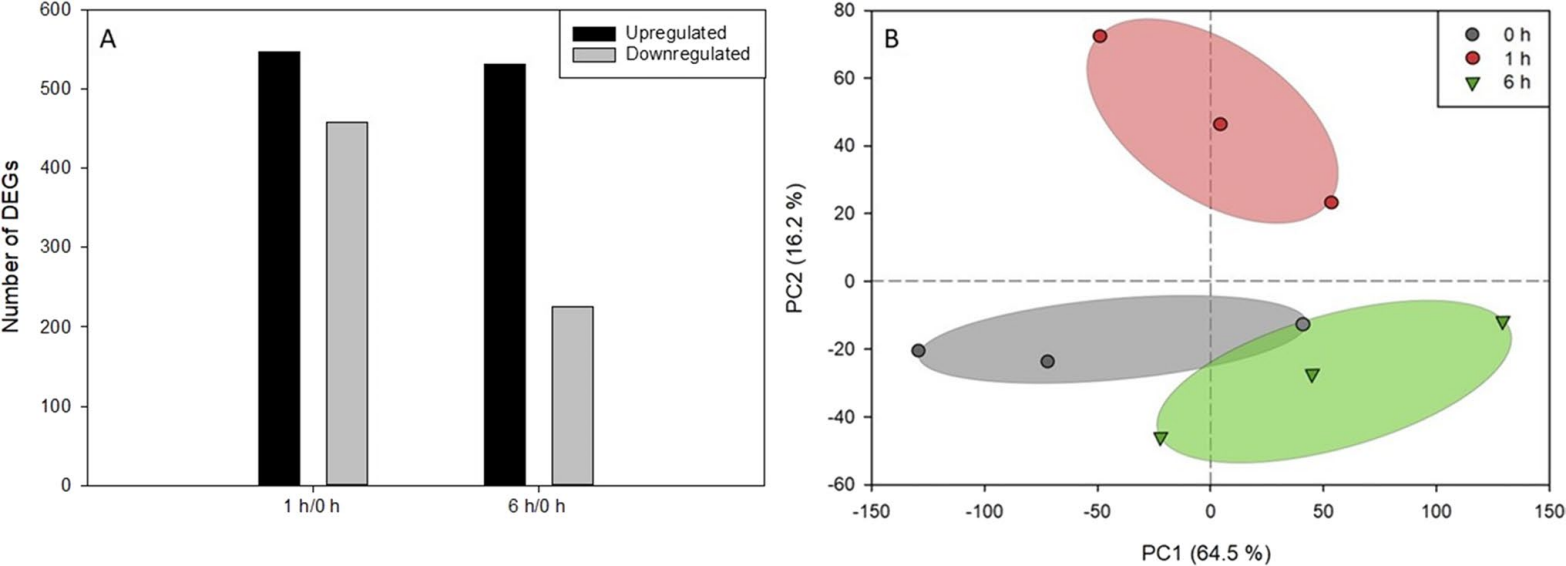
Overall differentiation of *T. chuii* transcriptome. **A** Number of log2-normalized significant (*p* < 0.05) transcripts with affected expression levels between different treatment comparisons (*n* = 1005). **B** Principal component analysis of transcripts following exposure to 0.5-mM H_2_O_2_. For a given treatment, each biological replicate is shown separately (*n* = 3). The dashed line separates the upper positive quartile of PC1

When comparing the DEGs of *T. chuii* cultures exposed to 0.5-mM H_2_O_2_ for 1 h to the control growth conditions, an extensive transcriptomic reprograming, affecting several cellular processes, was observed. A total of 1005 DEGs were reported (differential expression adjusted *p* < 0.05), with 547 being up-regulated, and 458 down-regulated (Fig. 2A). Interestingly, the prolonged exposure of the microalgal cells to the stressor for 6 h resulted in less profound effects, as 756 DEGs were identified, including 531 up-regulated, and 225 down-regulated (Fig. 2A). A principal component analysis (PCA) revealed a tight grouping of the studied transcriptomes (Fig. 2B). By plotting PC1 and PC2, representing a total contribution of 80.7% to the total variability, biological replicates for every treatment were clearly grouped (Fig. 2B). In the Euclidean space defined by PC1 and PC2, transcripts of 1-h exposure and 6-h exposure were placed on the longest route, while the control was at an intermediate position (Fig. 2B).

When focusing the analysis on specific metabolic biochemical processes (Supplemental Fig. S6), a wide array of transcription responses was observed, as inferred from the mapping of the identified differentially expressed genes (DEGs). Nine genes participating in purine metabolism (map00230) were found to be significantly down-regulated at 1 h, while four were up-regulated at 6 h and six were down-regulated. In relation to photosynthesis (map00195), ten genes were down-regulated at 1 h, and two were up-regulated at 6 h. Carbon metabolism (map01200) displayed 15 genes down-regulated at 1 h, eight down-regulated at 6 h, and four up-regulated at 6 h. Genes associated with glycolysis (map00010) showed down-regulation, with seven at 1 h and two at 6 h. Biosynthesis of amino acids (map01230) exhibited significant down-regulated activity at 1 h, with 22 genes, and five at 6 h. Interestingly, biosynthesis of secondary metabolites (map01110) experienced a notable decrease, with 48 being down-regulated at 1 h and 18 at 6 h. These results highlighted the reduced metabolic activity observed at 1 h in several major pathways, as well as the attempted reactivation of photosynthesis and carbon metabolism at 6 h.

**Fig. 3:**
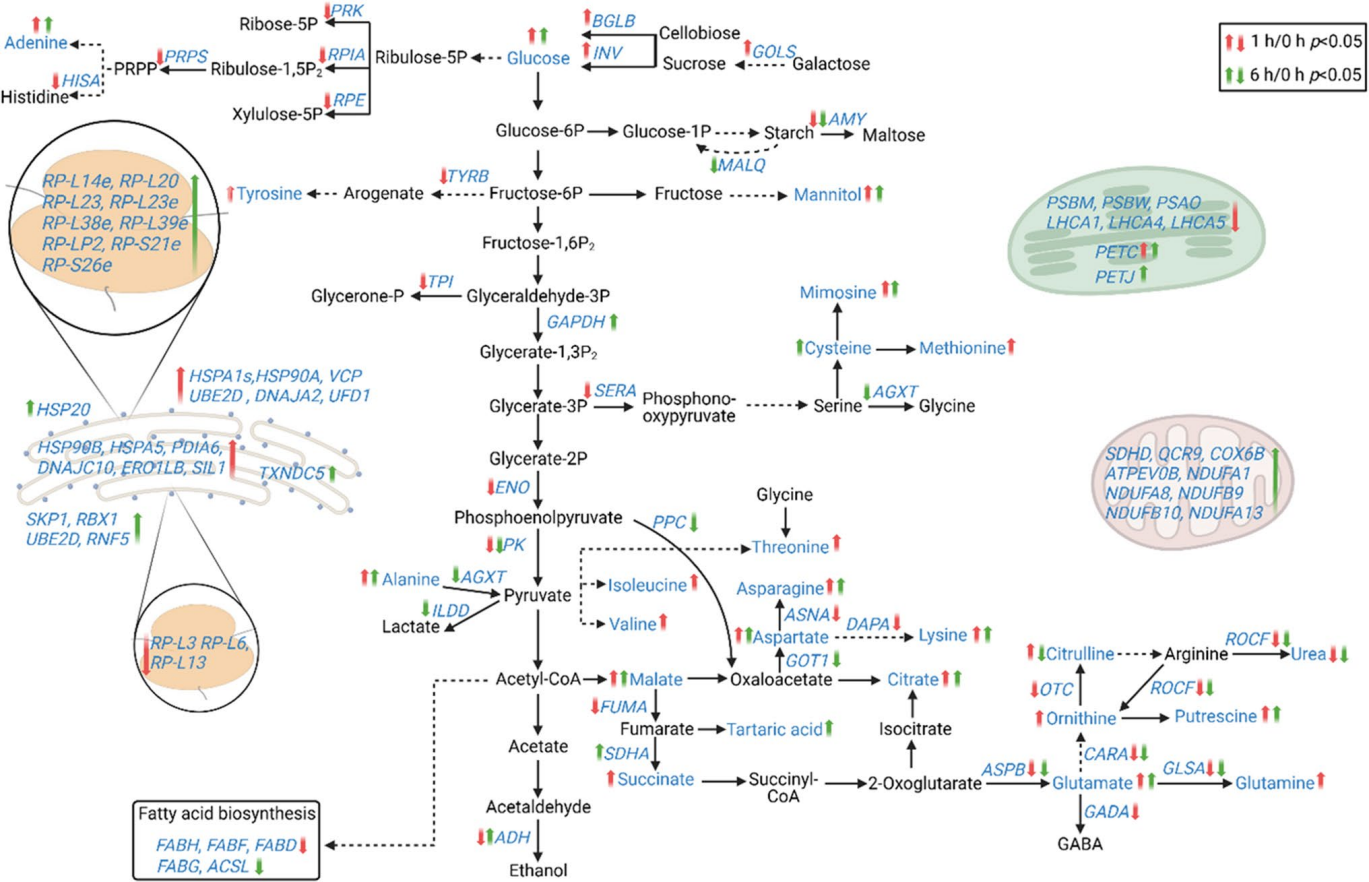
Global effect of different H_2_O_2_ concentrations on *T. chuii* transcriptome and metabolome. Pathway map representation of significant changes in transcriptome (*n* = 3 biologically independent experiments, *p* < 0.05) and metabolome (*n* = 6, three biologically independent experiments and two technical repeats each, *p* < 0.05). Genes and metabolites identified in this study are shown in blue. Metabolites in black are not measured in this study. Arrows next to genes or metabolites represent statistically significant up-regulation/accumulation and down-regulation/depletion. Red arrows indicate relative transcript or metabolite levels of *T. chuii* grown in the presence of 0.5-mM H_2_O_2_ for 1 h in comparison to corresponding levels of the same strain grown under control conditions (1 h/0 h). Green arrows indicate relative transcript or metabolite levels of *T. chuii* grown in presence of 0.5-mM H_2_O_2_ for 6 h in comparison to corresponding levels of the same strain grown under control conditions (6 h/0 h). The whole data set of fold changes and the statistical analysis are available in Supplemental Table S2

Delving into greater detail, the comparison of relative transcript levels between cells exposed to 0.5-mM H_2_O_2_ for 1 h and control cultures not exposed to the stressor (0 h) revealed an extensive early transcriptional reprogramming of the cells (Fig. 3, Table 2, Supplemental Table S2). Among the up-regulated DEGs, 15 played a role in protein processing in the endoplasmic reticulum (ER), including the genes for nucleotide exchange factor *SIL1,* DnaJ heat shock protein family (Hsp40) member C10 (*DNAJC10*)*,* heat shock protein family A (Hsp70) member 5 (*HSPA5*), and heat shock protein 90 β (*HSP90B*)*,* all of them involved in protein recognition by luminal chaperons, displaying 3-, 3-, 2.3-, and 1.8-fold higher levels than in the control, respectively. In addition, endoplasmic reticulum oxidoreductase 1 β (*ERO1LB*) and protein disulfide isomerase family A member 6 (*PDIA6*) transcripts, both responsible for protein targeting in the ER, were found significantly up-regulated. Several transcripts coding for the ubiquitin ligase complex and the subsequent ER-associated degradation towards proteasome were also up-regulated, including heat shock protein family (Hsp70) member 1 (*HSPA1s*), ubiquitin conjugating enzyme E2 D (*UBE2D*) and heat shock protein 90 α (*HSP90A*) genes which showed a 2.5-, 1.2-, and 4.9-fold increased transcript accumulation. Furthermore, genes related to peroxisome function were significantly up-regulated, including those for 3-hydroxymethyl-3-methylglutaryl-CoA lyase (*HMGCL*) and mitochondrial inner membrane protein (*MPV17*) (2.8- and 2.3-fold, respectively). H_2_O_2_-induced transcripts also included invertase (*INV*) and β-glucosidase (*BGLB*) genes involved in starch and sucrose metabolism. Exposure to H_2_O_2_ demonstrated a beneficial effect on galactose metabolism, as evidenced by the induction of three transcripts, notably including an impressive 8.6-fold up-regulation of galactinol synthase gene (*GOLS*) expression. Moreover, among the induced DEGs were the genes for ring finger protein 41 (*RNF41*), charged multivesicular body protein 1 (*CHMP1*), vacuolar protein sorting protein 29 (*VPS29*), and IST1 factor associated with ESCRT-III (*IST1*), all of them playing pivotal roles in the mechanism of endocytosis. The expression of genes associated with the biosynthesis of secondary metabolites exhibited a substantial increase, while the biosynthesis of cofactors and the MAPK signaling pathway were also positively affected.

**Table 2:**
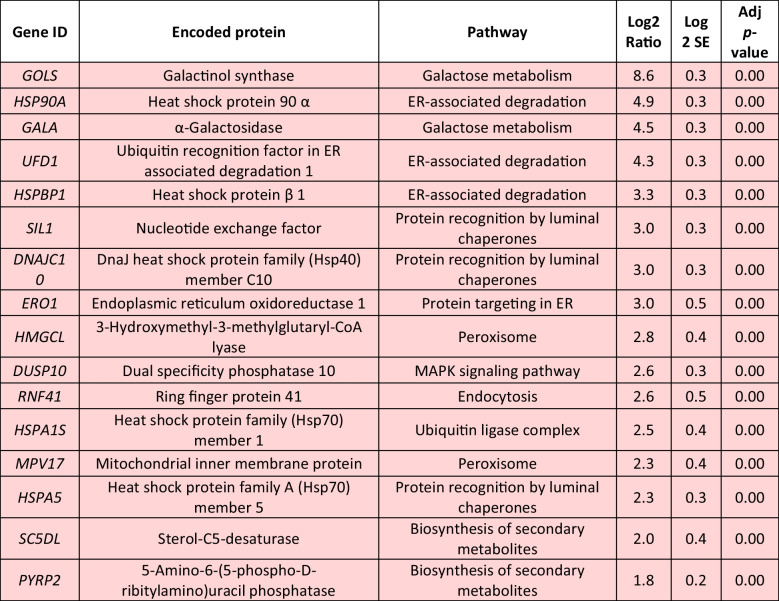

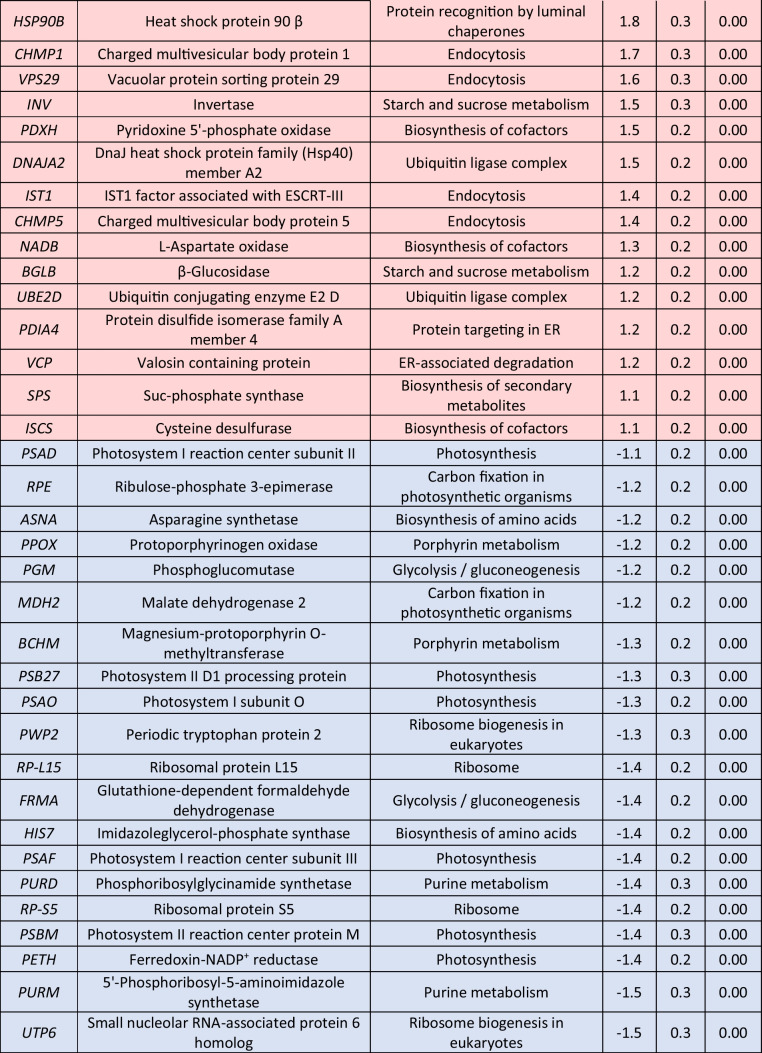

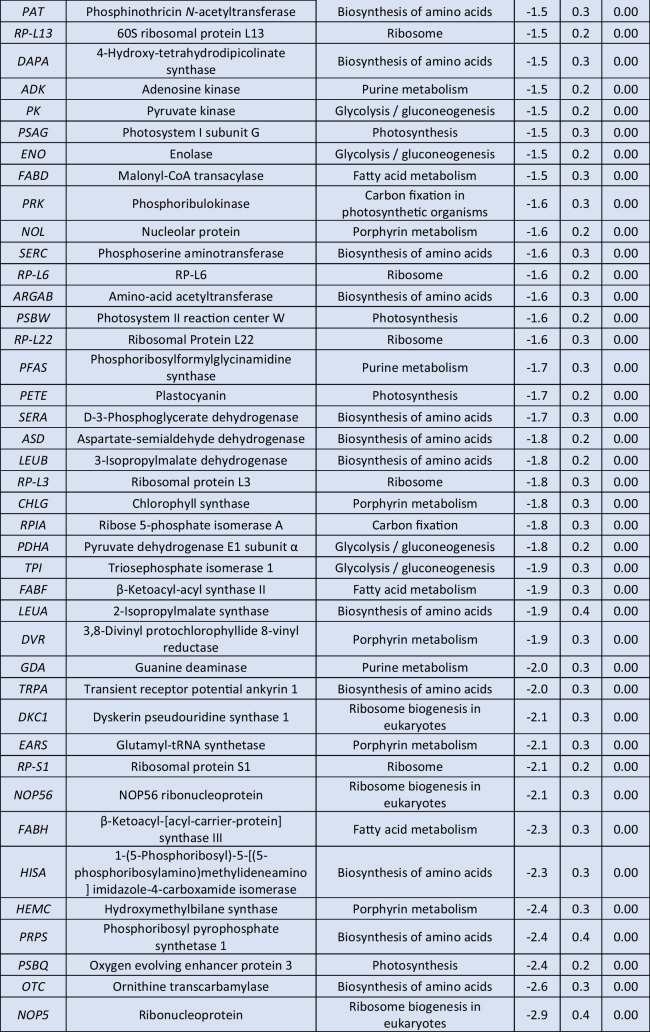
Log_2_-normalized fold changes of well-annotated transcripts with significantly affected expression levels (*p* < 0.05) between 1 and 0 h.^1^

^1^For each treatment, ratios were calculated from the average of three biological repeats (*n* = 3)

Red color indicates significantly upregulated DEGs, blue color indicates significantly downregulated DEGs

Exposure of the microalgal cells to 0.5-mM H_2_O_2_ for 1 h resulted in a significant down-regulation of several transcripts involved in central cellular processes. Carbon metabolism was profoundly affected, as 15 of the down-regulated DEGs participate in this process. These included the genes for the key factors of glycolysis/gluconeogenesis pyruvate kinase (*PK*) and enolase (*ENO*), which were both found to be 1.5-fold down-regulated, and other genes like for glutathione-dependent formaldehyde dehydrogenase (*FRMA*), pyruvate dehydrogenase E1 subunit α (*PDHA*) and triosephosphate isomerase 1 (*TPI*). The down-regulation of carbon metabolism was also depicted in the reduction of carbon fixation, as indicated by the reduced expression levels of several transcripts, including a 1.8-fold decrease observed for the gene for ribose 5-phosphate isomerase A (*RPIA*). Regarding photosynthesis, the impact of the stress was acute, as four genes coding for subunits of PSI (photosystem I) and five genes coding for subunits of PSII were down-regulated, with expression levels of oxygen evolving enhancer protein 3 (*PSBQ*) gene reduced by 2.4-fold. Expression of plastocyanin-coding gene *PETE* and ferredoxin-NADP^+^ reductase gene *PETH* was also downregulated by 1.7-fold and 1.4-fold, respectively. In addition, the expression of several transcripts coding for the light harvesting complexes was down-regulated. The transcription of genes involved in fatty acid metabolism was also negatively affected by the exposure to H_2_O_2_, as lower expression levels of the genes for malonyl CoA-acyl carrier protein transacylase (*FABD*), β-ketoacyl-acyl carrier protein synthase III (*FABH*), and β-ketoacyl-acyl carrier protein (ACP) synthase II (*FABF)* were observed. Biosynthesis of amino acids was severely affected, as 22 down-regulated DEGs were identified. Among them, the most profound down-regulation in their respective transcript levels was observed for the genes for ornithine transcarbamylase (*OTC*)*,* phosphoribosyl pyrophosphate synthetase 1 (*PRPS*), and 1-(5-phosphoribosyl)-5-[(5-phosphoribosylamino) methylideneamino] imidazole-4-carboxamide isomerase (*HISA*)*,* exhibiting 2.6-, 2.4-, and 2.3-fold lower transcripts. Several genes coding for ribosome structural proteins were also found to be down-regulated, including *RP-L3*, *RP-L6*, and *RP-L13*, as well as five genes involved in ribosome biogenesis. Exposure to 0.5-mM H_2_O_2_ for 1 h also had a negative impact on other pathways, as purine and porphyrin metabolism.

The prolonged exposure to 0.5-mM H_2_O_2_ for 6 h resulted in a lower number of identified DEGs (Table 3, Supplemental Table S2). Among the up-regulated DEGs, nine were associated with oxidative phosphorylation, including the twofold up-regulated genes for NADH ubiquinone oxidoreductase subunits A1 and A8 (*NDUFA1*, *NDUFA8*). In addition, the genes for cytochrome c6 (*PETJ*) and Rieske iron-sulfur protein (*PETC*), whose products are both implicated in photosynthesis, were found to be more than twofold up-regulated in comparison with the control. Multiple transcripts involved in carbon metabolism were positively affected, among which the gene for glyceraldehyde 3-phosphate dehydrogenase (*GAPDH*) exhibited a notable up-regulation of at least twofold. Furthermore, ribosome formation was strongly enhanced, as 16 genes encoding structural proteins of both small and large ribosomal subunits were significantly up-regulated, including *RP-L38e*, *RP-L35e*, and *RP-L23e* transcripts, all exhibiting at least a 2.1-fold increase. Thioredoxin domain containing 5 (*TXNDC5*) transcripts, responsible for protein targeting to the ER, were found to be more than twofold up-regulated, while heat shock protein 20 (*HSP20*) transcripts, involved in the ER-associated degradation, were also up-regulated. The ubiquitin ligase complex was also affected with S-phase kinase associated protein 1 (*SKP1*), ring-box 1 (*RBX1*), ring finger protein 5 (*RNF5*), and *UBE2D* genes being slightly overexpressed. Expression levels of several genes participating in nucleotide excision repair were up-regulated, including the genes for RNA polymerase II (*RPABC2*) and proliferating cell nuclear antigen (*PCNA*) with observed 2.5- and 1.8-fold higher transcript levels, respectively. Other up-regulated DEGs were coding for proteasome subunits.

**Table 3:**
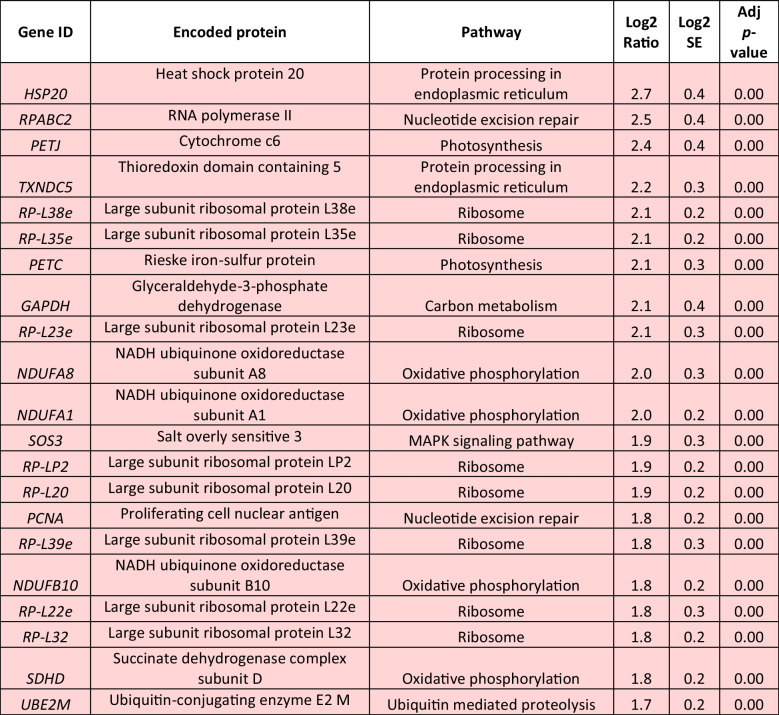

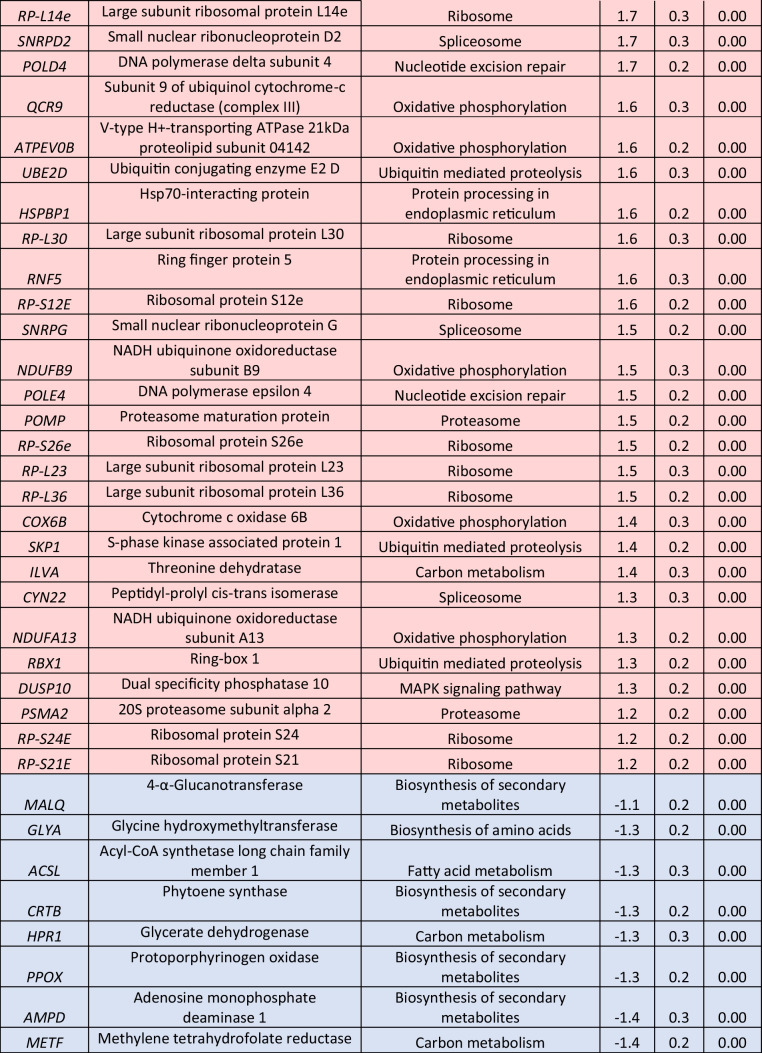

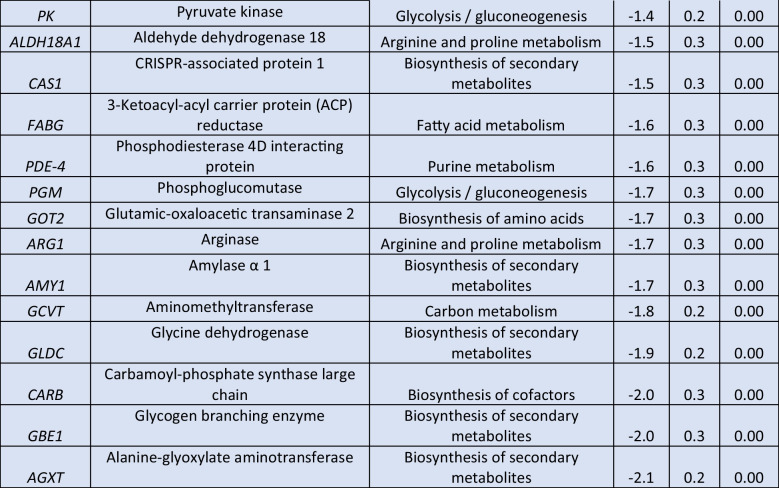
Log_2_-normalized fold changes of well-annotated transcripts with significantly affected expression levels (*p* < 0.05).^1^

^1^For each treatment, ratios were calculated from the average of three biological repeats (*n* = 3)

Red color indicates significantly upregulated DEGs, blue color indicates significantly downregulated DEGs

In contrast to previous results, exposure to 0.5-mM H_2_O_2_ for 6 h led to the down-regulation of several biosynthetic pathways. Specifically, 18 of the identified down-regulated DEGs participate in biosynthesis of secondary metabolites, with genes for glycogen branching enzyme (*GBE1*) and alanine-glyoxylate aminotransferase (*AGXT*) exhibiting the most significant decrease, with respective reduction of 2.0- and 2.1-fold. In addition, down-regulation was observed in the expression levels of genes involved in amino acid metabolism, including alanine, aspartate, and glutamate, depicted in the significantly reduced levels of glutamic-oxaloacetic transaminase 2 (*GOT2*) transcripts. Moreover, down-regulation was observed in three transcripts involved in arginine and proline metabolism, as well as in DEGs participating in the biosynthesis of cofactors, including the twofold decrease of the gene for carbamoyl-phosphate synthase large chain (*CARB*). Similarly, the gene for pyruvate kinase (*PK*), which plays a key role in glycolysis, and the gene for phosphoglucomutase (*PGM*), were identified among the down-regulated DEGs. In addition, down-regulation was observed in fatty acid metabolism, as 3-ketoacyl-acyl carrier protein (ACP) reductase (*FABG*) and acyl-CoA synthetase long chain family member 1 (*ACSL*) transcript levels were 1.6- and 1.3-fold lower compared to the control treatment, respectively. Photorespiration exhibited a significant decrease, as evidenced by the down-regulation of several transcripts involved in this metabolic process, including the aminomethyltransferase gene *GCVT*. Among the identified down-regulated DEGs were also several participating in purine metabolism, including phosphodiesterase 4D interacting protein (*PDE-4*).

### Global metabolomic responses upon H_2_O_2_-induced oxidative stress

**Fig. 4:**
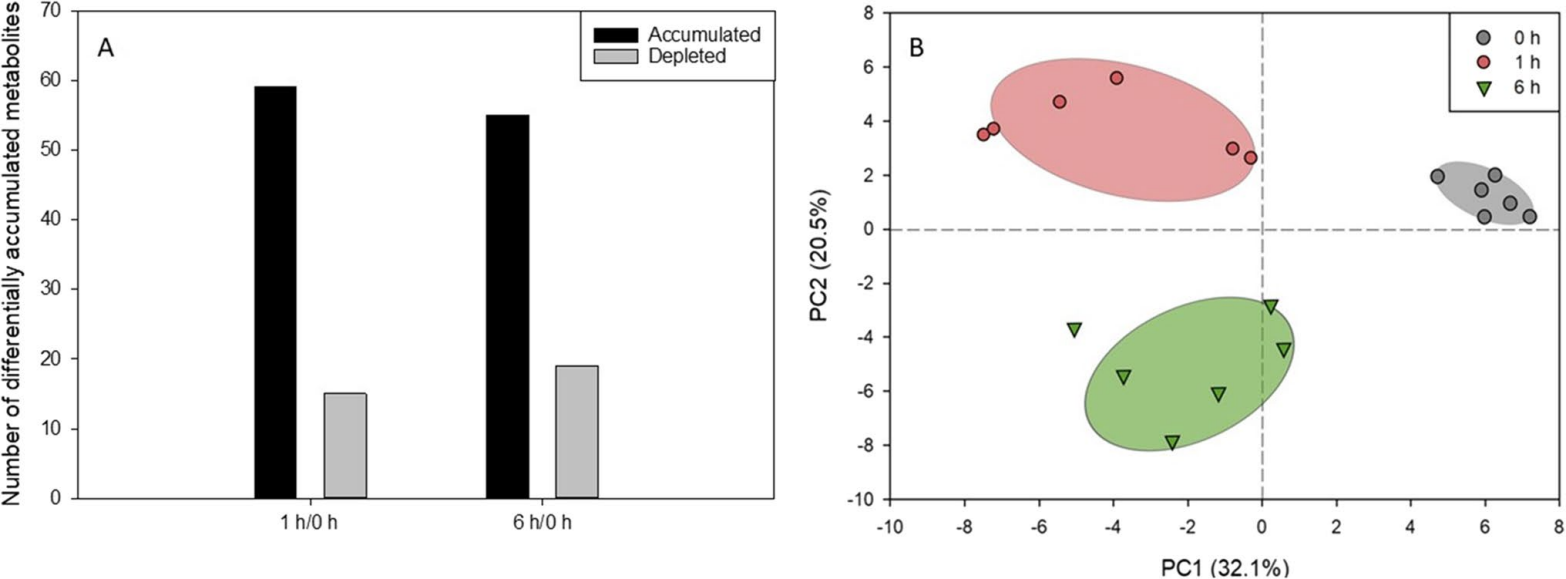
Overall differentiation of *T. chuii* metabolome. **A** Number of metabolites with affected content between different treatment comparisons (*n* = 74). **B** PLS-DA analysis of metabolites following exposure to 0.5-mM H_2_O_2_. For a given treatment, each biological and technical replicate is shown separately (*n* = 6). The dashed line separates the upper positive quartile of PC1

To gain a better understanding of how the observed transcriptional reprogramming during H_2_O_2_-induced oxidative stress reflects on the overall metabolic homeostasis of the cell, we utilized an untargeted metabolomic approach with a GC–MS platform. This analysis enabled the identification and relative quantification of over 70 metabolites, including amino acids, sugars, organic acids, and nitrogen-containing compounds (Fig. 4A, Supplemental Table S3). When comparing the metabolite content of cultures exposed to 0.5-mM H_2_O_2_ for 1 h with the content in control cells, we were able to identify 41 metabolites showing a significant increase in their content, while only three exhibited reduced content under stress conditions. Similarly, comparing the content after 6 h of exposure, we observed a significant increase in 28 metabolites, while five identified metabolites showed reduced accumulation under stress conditions. To study the overall response of the metabolome, we performed PLS-DA analysis. This analysis effectively demonstrated the grouping and separation of metabolites from all treatments, with the metabolites from control cells being the farthest from those of both 1-h and 6-h exposures (Fig. 4B). This pattern starkly resembled with the respective PLS-DA transcriptome analysis.

Comparison of the metabolomic profiles of *T. chuii* cultures exposed to 0.5-mM H_2_O_2_ for 1 h revealed several differentially accumulated metabolites (Fig. 3, Table 4). Amino acids were strongly affected, as 13 of them showed increased contents at these early stages of the cell responses, including methionine, valine, and asparagine, which showed 10.7-, 3.8-, and 3.8-fold increased metabolite content, respectively, when compared to the control. In response to oxidative stress, the content of eight organic acids increased. Citric acid and malic acid showed the highest accumulation with a 7.5- and 5.9-fold increase, respectively. Additionally, glucose exhibited a significant increase (5.7-fold). Among the nitrogen-containing compounds, adenine exhibited a threefold increase in accumulation, while putrescine increased by 1.5-fold. Conversely, the content of urea was threefold lower compared to control cultures. Furthermore, polyol content, like mannitol, was increased in cultures exposed to 0.5-mM H_2_O_2_ for 1 h.

**Table 4:**
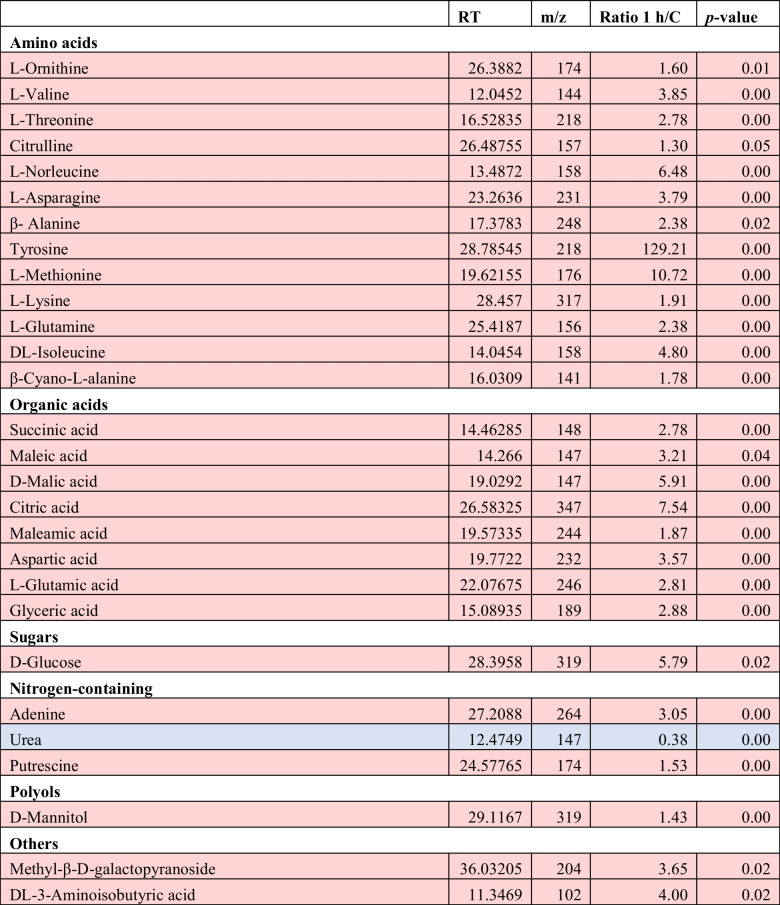
Relative response ratios of identified metabolites with statistically significant differences (*p* < 0.05, ANOVA) for 1 h and 0 h.^1^

^1^For each treatment, ratios were calculated from the average of three biological and two experimental repeats for each (*n* = 6)

Red color indicates metabolites with significantly accumulated content, blue color indicates metabolites with significantly depleted content

Interestingly, the effects of 0.5-mM H_2_O_2_ exposure for 6 h on *T. chuii* metabolome were less pronounced (Fig. 3, Table 5). Regarding the amino acids, asparagine, alanine, and lysine exhibited a 2.6-, 3.4-, and 1.8-fold increase in stressed cells, whereas citrulline content was 1.5-fold lower. Among the seven significantly affected organic acids, citric acid displayed the highest accumulation with an 11.7-fold change, while oxalic acid was the only one exhibiting a decrease (fivefold) in content. In the case of sugars and polyols, glucose content showed a significant increase with a 14.2-fold change, and mannitol also increased by 1.5-fold. Furthermore, four nitrogen-containing metabolites were found to accumulate, with adenine, methylalanine, putrescine, and spermidine showing a 2.5-, 4-, 1.6-, and 3.2-fold increase, respectively. Conversely, urea content experienced a substantial decrease in stressed cells with a 12-fold change. Additionally, the total content of organic acids was higher after exposure to H_2_O_2_. Specifically, the highest content was observed after 1 h, gradually decreasing in subsequent hours, while remaining at elevated levels compared to the 0-h control (Supplemental Table S3).

**Table 5:**
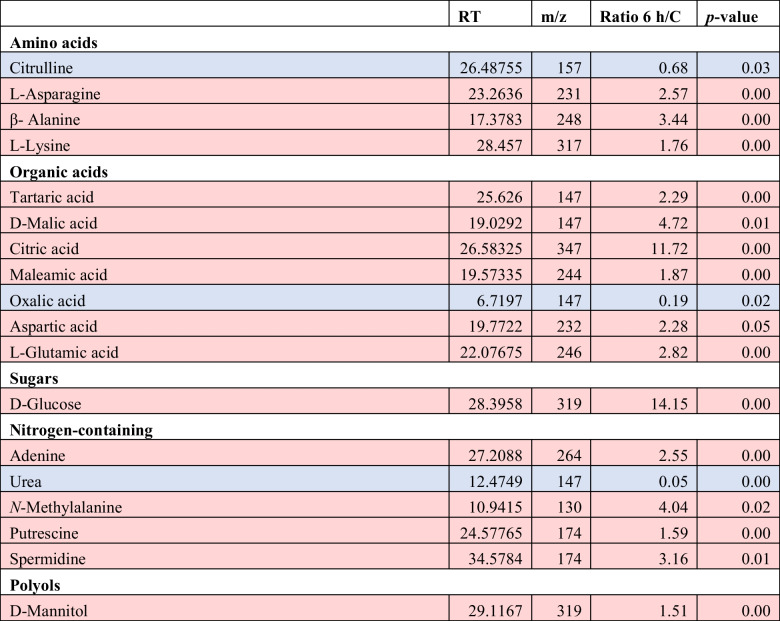
Relative response ratios of identified metabolites with statistically significant differences (*p* < 0.05, ANOVA) for 6 h and 0 h.^1^

^1^For each treatment, ratios were calculated from the average of three biological and two experimental repeats for each (*n* = 6)

Red color indicates metabolites with significantly accumulated content, blue color indicates metabolites with significantly depleted content

## Discussion

Microalgae are continually exposed to abiotic stresses as a consequence of the constantly changing habitat (Odjadjare et al. 2017). In order to ensure their successful adaptation and survival, they have evolved a multitude of adaptations, which differ among species, due to their unique morphological, physiological, and genomic characteristics (Barone et al. 2021). In the present study, the effects of H_2_O_2_-induced oxidative stress on the microalga *T. chuii* have been examined, providing new insights on its molecular and biochemical adaptation mechanisms.

### Altered physiology strongly dictates a metabolic reprogramming upon oxidative stress in T. chuii

As indicated by the observed cell viability, all used H_2_O_2_ concentrations led to induction of oxidative stress to *T. chuii* cultures within a relatively short period of time (Fig. 1). The addition of 0.5-mM H_2_O_2_ affects the cells’ viability significantly, although a tolerance antioxidant mechanism appears to be activated. Higher concentrations led to an immediate dramatic reduction in cell viability and the eventual collapse of the cultures. This quick reaction to H_2_O_2_ corroborates with the sensing ability of green microalga *C. reinhardtii*, whereas a higher H_2_O_2_ dosage up to 2-mM H_2_O_2_ was required for reducing cell viability (Koletti et al. 2022). This observation highlights the differences among green microalgal cells, with *T. chuii* being more sensitive to oxidative stress.

As our results revealed, early responses of green microalgae to oxidative stress include the significant reduction of photosynthesis and the total carotenoids content, as well as an observed slight decline on total lipids, total phenolic content, and chlorophylls. The utilization of carotenoids has been shown as an antioxidative strategy of green microalgae in the past, able to cease oxidation reactions, revealing their crucial role as an early antioxidative reaction in *T. chuii* (Gauthier et al. 2020; Sansone and Brunet 2019). In addition, the reprogramming of their metabolism towards ROS tolerance corroborated the previous studies (Chen et al. 2020). The exposure of cells to 0.5-mM H_2_O_2_ indicated that the early microalgal response to stress occurs within the 1st hour, resulting in the observed metabolic adaptations after long term exposure, although photosynthetic capacity of PSII keeps declining. This is evident from the RNAseq analysis whereas a higher number of DEGs for the 1 h vs 0 h comparison than the 6 h vs 0 h were reported, suggesting the initial global transcriptomic responses occurred within the 1st hour (Fig. 2). One-hour exposure treatment showed a totally different transcriptome profile, while 6-h profile was more similar to the control group (Fig. 2). This could suggest a fast-response metabolic reprogramming mechanism in green microalgae responsible form the initial adaptation reactions upon externally induced oxidative stress. Global untargeted metabolomic analysis also revealed that the majority of the significantly affected metabolite profiles corresponded to the early stages of H_2_O_2_-induced oxidative stress (Fig. 3). This observation is consistent with previous work in *C. reinhardtii*, in which microalgal cultures decompose H_2_O_2_ rapidly, within 4 h, and persistent transcriptional responses to oxidative stress occur as early as the first hour of exposure to the stressor (Blaby et al. 2015).

### T. chuii attenuates carbon fixation upon short-term oxidative stress

The induction of short-term oxidative stress in *T. chuii* was shown to significantly affect several cellular processes. Specifically, expression levels of chaperone genes in the ER were increased, as also shown in previous reports (Ma et al. 2020). A typical example is the increased transcript levels of heat shock proteins (HSPs) which are well described molecular chaperones responding to various environmental niches, having a similar function in microalgal cells as well (Åkerfelt et al. 2010; Lee et al. 2014). Similarly, in *C. reinhardtii* cells exposed to 2-mM H_2_O_2_ for 1 h, the up-regulation of several HSPs was observed (Koletti et al. 2022). Several, DNAJ domain-containing heat sock transcripts, including *DNAJA2* and *DNAJC10*, were found up-regulated, which belong to the four families of evolutionarily conserved H_2_O_2_-sensitive eukaryotic proteins among all kingdoms (Vandenbroucke et al. 2008). In addition, genes such as *HSPA1s* and *UBE2D* involved in targeting proteins for degradation, by catalyzing the attachment of ubiquitin in a lysine residue, were found up-regulated, possibly indicating an increase in protein recycling in order to meet with a decreased availability in amino acids (Ma et al. 2020). Furthermore, upon 1-h exposure to H_2_O_2_, peroxisome-associated transcripts like from gene *MPV17* were significantly up-regulated, depicting the activation of the cell integral ROS detoxification (Kao et al. 2018).

Interestingly, in parallel to the observed increased transcription of protein degradeosome genes, a decrease in the expression levels of genes, including *RP-L3* and *RP-L6*, coding for ribosomal subunits was observed upon 1 h of H_2_O_2_-induced oxidative stress. In addition, our results revealed that the accumulation of 22 transcripts involved in the biosynthesis of basic amino acids, including from the genes for tyrosine aminotransferase (*TYRB*), *HISA*, and 4-hydroxy-tetrahydrodipicolinate synthase (*DAPA*), significantly decreased. In contrast, our global untargeted metabolomic analysis revealed that the content of several amino acids, including tyrosine, methionine, asparagine, and lysine, was significantly higher upon H_2_O_2_-induced oxidative stress. In the case of methionine, its increased content could also account for its protein-protective role upon oxidative stress (Luo and Levine 2009). In total, our results provide support to the hypothesis that protein degradation and the reduction of protein synthesis, which subsequently leads to amino acid accumulation, could represent a first line of defense upon oxidative stress allowing the microalgal cells to fuel gluconeogenesis (Blaby et al. 2015). Evidence of protein degradation is also provided by observed accumulation of various nitrogen-containing compounds, such as nitrogen-containing mimosine, which was detected only in the presence of stressor and has been shown to act as antioxidant under abiotic stress the plant *Leucaena leucocephala* (Honda and Borthakur 2021). Similarly, the accumulation of adenine is also known to trigger stress tolerance (Sukrong et al. 2012). The increased content of citrulline and ornithine is also in agreement with previous results, with the former being an indicator of tolerance to several abiotic stresses in *Cucumis melo*, while the latter has been shown to accumulate in *Chlorella vulgaris* during cadmium stress (Kusvuran et al. 2013). Finally, putrescine is one of the polyamines found in microalgae and known to participate in several molecular mechanisms employed by the cell in order to mitigate the adverse impacts from the environment (Xu et al. 2021).

Overall, H_2_O_2_-induced oxidative stress had a strong effect on carbon and energy flow and metabolism in *T. chuii*, shown also by the slight reduction of total sugars. Among the most biochemically profound adaptations, *PK* transcripts, coding for the key glycolytic enzyme pyruvate kinase, were found to be significantly down-regulated upon 1 h of exposure to H_2_O_2_. Similarly, in the *Chlorophyta Ulva compressa* pyruvate kinase was inhibited as a consequence of copper-induced oxidative stress (Laporte et al. 2020). The reduced energy production via glycolysis early upon exposure to H_2_O_2_ is exacerbated by a decrease in the expression of *ENO* transcripts, which encode enolase, the pre-pyruvate kinase enzyme, and several other genes participating in the glycolysis pathway. In general, glucose can act as an important antioxidative metabolite due to its involvement in NADPH production (Cherkas et al. 2020). It also owns a pivotal role in stress signaling and stress acclimation in plants. The increased glucose content enhances tolerance to ROS-related abiotic stress, as previously reported in plants exposed to high salinity (Sanchez et al. 2007; Skliros et al. 2018). It could be that glucose accumulation can have a multilevel role in ROS-stressed photosynthetic organisms when carbon fixation is attenuated, as depicted in the present study by the down-regulation of *RPIA* coding for the ribose 5-phosphate isomerase A. In addition, starch metabolism was up-regulated due to oxidative stress, a stress response previously also reported to several microalgae (Li et al. 2015). In addition, 1-h exposure to H_2_O_2_ led to the down-regulation of several genes coding for proteins of the light-harvesting complex, and the down-regulation of *PSBM*, *PSBW*, and *PSAO* of the PSI and PSII, alongside with the decrease of the maximal PSII quantum yield. These observations are in line with the reported limited rate of photosynthesis in *C. reinhardtii* as a result of abiotic stress (Pillai et al. 2014; Koletti et al. 2022) and corroborate well with the shift towards intracellular gluconeogenesis as energy source, possibly also utilizing the excess of free amino acids resulting from protein degradation. *GOLS* transcripts, participating in galactose metabolism were highly up-regulated, a finding in line with the strong increase of this pathway in *C. vulgaris* as a response to free ammonia stress (Dai et al. 2023). Also, the observed up-regulation of transcript expression levels associated with endocytosis is a common response of green microalgae to short-term stress (Barten et al. 2022).

### Some photosynthesis-related genes of T. chuii are up-regulated upon long-term oxidative stress exposure

The prolonged exposure to the stressor led to distinct long-term molecular adaptation mechanisms to externally induced oxidative stress in *T. chuii*. In general, the long-term oxidative stress had a profound effect on antioxidant potential, total phenolic content, total lipids, total sugars, and total carotenoids, indicating a global reprogram towards utilization of potent antioxidant properties of secondary metabolites and consumption of intracellular carbon (in the form of sugars and lipids) to withstand the stress, which appear depleted over time (Goiris et al. 2012). At the same time, PSI-related gene *PETJ* transcripts were significantly increased, while transcripts associated with photorespiration were significantly decreased. Additionally, enrichment analysis revealed a cascade shut down of the photosynthesis pathway under 6-h exposure to the stress. Contrary to these results, the expression levels of PSII-related gene *PETC* were found significantly increased, although measurements of PSII efficiency kept declining. Based on these results, it is plausible to assume that on both the molecular and metabolic levels, cells with depleted intracellular carbon and fatty acids are trying to merely reset their photosynthetic capacity and subsequently carbon fixation, but possibly the observed effect of ROS on protein synthesis act as a drawback towards achieving their initial photosynthetic homeostasis (Khorobrykh et al. 2020). Due to a possible convergence of H_2_O_2_-signals in chloroplasts and mitochondria (Dourmap et al. 2020), various transcripts associated with oxidative phosphorylation were up-regulated. After facing 6 h of oxidative stress, *T. chuii* endeavors to resume carbon fixation, leading to increased expression levels of the glycolysis-participating *GAPDH*. However, this progress is hindered by the down-regulation of *PK* and *PGM*. Interestingly, while expression of *FUMA* coding for fumarase A was down-regulated, the contents of succinic, malic, and citric acids, all participating in the tricarboxylic acid (TCA) cycle, were significantly increased compared to the control. It is possible that after 6-h exposure to H_2_O_2_-induced oxidative stress, the gluconeogenesis capacity of microalgal cells is depleted and microalgal cells are actively trying to revert their energy metabolism towards photosynthesis and glycolysis for survival.

Additionally, the induction of long-term oxidative stress in the *T. chuii* significantly affected transcripts related to the ER processes, as expression levels of heat shock mRNAs were found to be significantly increased upon this treatment as well. In contrast to the observed effects after 1-h exposure to external H_2_O_2_, long-term exposure to H_2_O_2_ led to increased expression of genes coding for ribosomal subunits, also indicating the differential cellular responses to prolonged oxidative stress. Nitrogen-containing putrescine, adenine, and spermidine content was also higher after 6 h to H_2_O_2_ exposure, with the later previously suggested to exhibit an extensive protective effect against oxidative damage in the microalga *Chlorella* sp. (Wang et al. 2020).

Interestingly, a consistent adaptation response in both long- and short-term oxidative stress in *T. chuii* cells was the significant increase in nitrogen-containing compounds content. This is a response often observed as a consequence of exposure to environmental stresses and occurs in the cells' attempt to detoxify the excess of free ammonia (Rare 1990). Furthermore, the significant reduction of urea in the presence of H_2_O_2_ can be interpreted as a result of the accumulation of all the aforementioned nitrogen-containing compounds, as urea is both considered an efficient nitrogen source (Witte 2011) and a scavenging agent of ROS in higher organisms. Green microalgae can efficiently degrade urea and subsequently utilize it as a source of carbon and nitrogen, as observed in *C. vulgaris* (Barros et al. 2017). However, compared to 1-h H_2_O_2_-induced oxidative stress, the amino acids content was depleted in 6 h. This could depict the cellular attempt to revert to the normal metabolic homeostasis at this time point. Finally, organic acids are known to be the major carbon source upon abiotic stresses in higher plants. Here we observed a depletion of organic acids from 1 to 6 h except from citric acid, which is well described that can confer abiotic stress tolerance in microalgae and plants (Li et al. 2013; Tahjib-Ul-Arif et al. 2021).

**Fig. 5:**
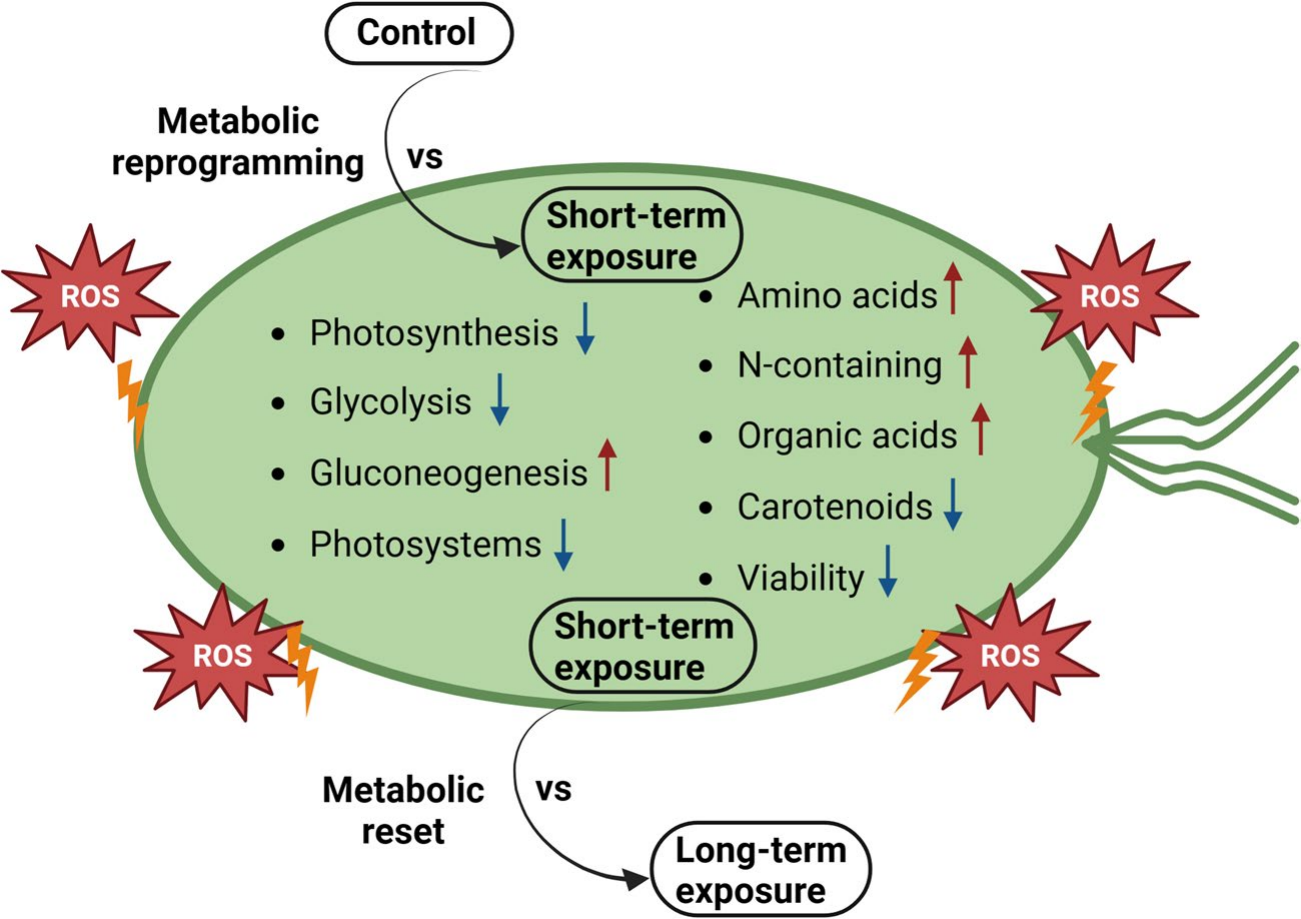
Schematic representation of main metabolic alterations taking place inside the cell during short-term exposure to oxidative stress. Red arrows depict upregulation, accumulation, and induced metabolic activity; blue arrows depict downregulation, depletion, and reduced metabolic activity

In summary, our results indicate that 0.5-mM H_2_O_2_-induced oxidative stress strongly affects *T. chuii* molecular and metabolic homeostasis, resulting in distinct short- and long-term adaptation mechanisms (Fig. 5). Within the first hour of exposure, cellular transcriptome and metabolome were shifted towards a stress survival mode, while during a long-term exposure of at least 6 h an adaptation attempt is observed aiming towards the maintenance of long-term metabolic homeostasis. Based on these observations, an intriguing hypothesis could be that *T. chuii* has developed a quick sense response for regulating a metabolic reprogramming against oxidative stress, but long exposure has detrimental effects in cellular physiology and protein malformation, requiring a more robust metabolic reprograming. This could constitute an evolutionary well-orchestrated adaptation strategy of green microalgae to short-term oxidizing conditions of their fast-changing microenvironment.

## Supplementary Information

Below is the link to the electronic supplementary material.

Supplementary file1 (PDF 674 KB)

Supplementary file2 (XLSX 145 KB)

## Data Availability

All data supporting the findings of this study are provided within the paper and its Supplemental information. We deposited all RNAseq data to the European Nucleotide Archive under accession number PRJEB61814.
