# Supplementary material for: Global omics study of Tetraselmis chuii reveals time-related metabolic adaptations upon oxidative stress
Source: Appl Microbiol Biotechnol. 2024 Jan 16;108(1):138. doi: 10.1007/s00253-023-12936-z (PMC10791844; doi:10.1007/s00253-023-12936-z)
Supplement: Supplementary file 1 — Supplementary file1 (PDF 674 KB) [file 253_2023_12936_MOESM1_ESM.pdf]

Applied Microbiology and Biotechnology

Supplemental Material

Global omics study of *Tetraselmis chuii* reveals time-related metabolic adaptations upon oxidative stress

Aikaterini Koletti[1], Dimitrios Skliros[1], Chrysanthi Kalloniati[2], Sofia Marka[1], Maria-Eleftheria Zografaki[1], Carlos Infante[3], Lalia Mantecón[3] and Emmanouil Flemetakis[1]*

[1]Department of Biotechnology, School of Applied Biology and Biotechnology, Agricultural University of Athens, Athens 11855, Greece

[2]Department of Marine Sciences, University of the Aegean, University Hill 81100, Mytilene, Greece

[3]Fitoplancton Marino, S.L., Dársena comercial s/n (Muelle pesquero), 11500 El Puerto de Santa María (Cádiz), Spain

*Correspondence: mflem@aua.gr

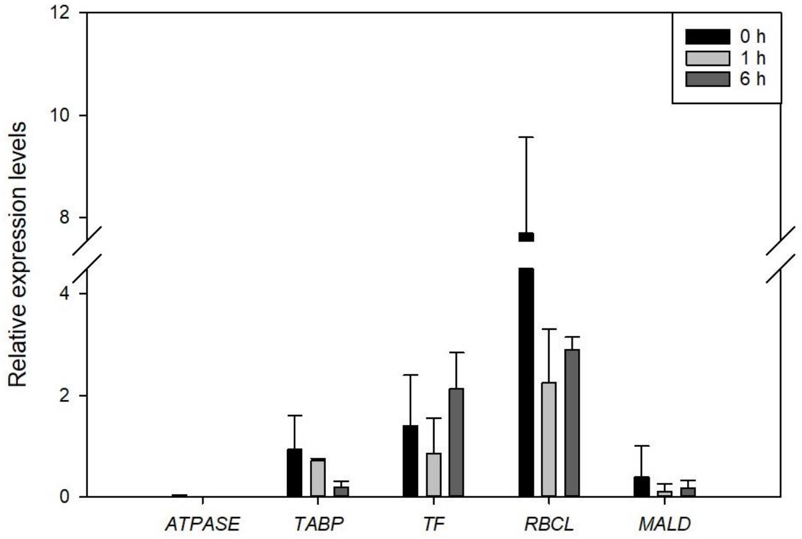

Figure S1: Relative expression levels of genes according to qPCR

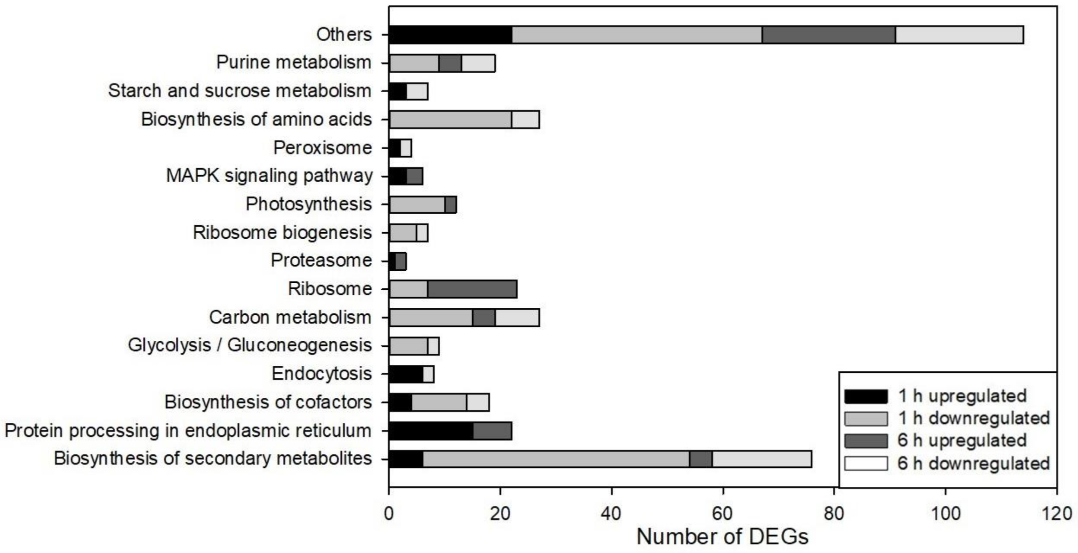

Figure S2: Number of DEGs annotated with KOs and mapped to pathways

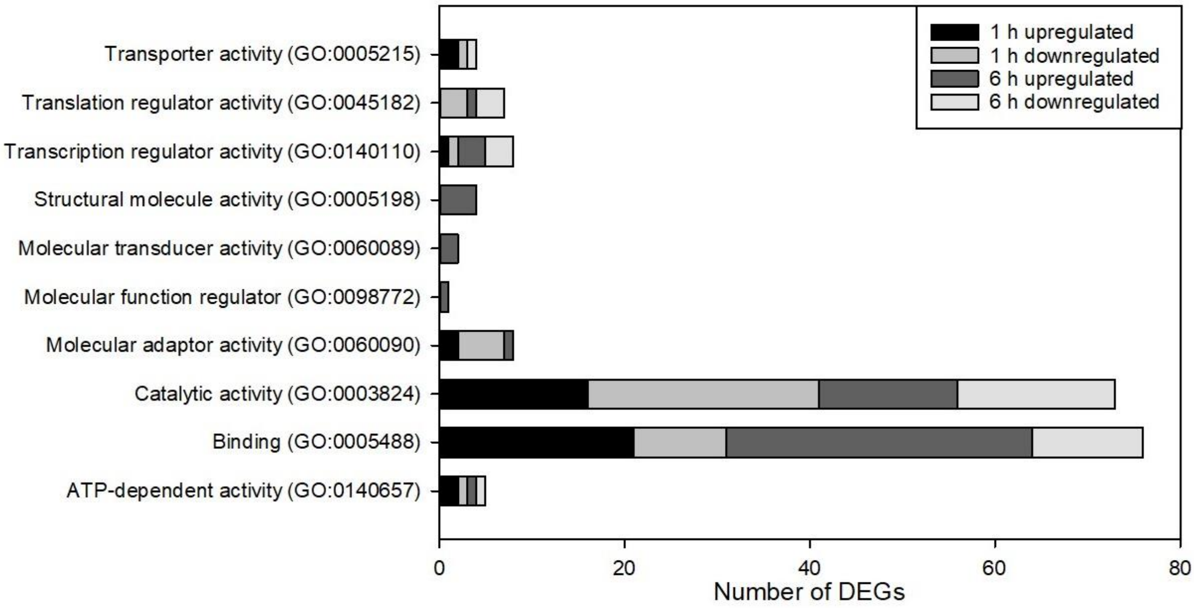

Figure S3: Number of DEGs classified based on GOs

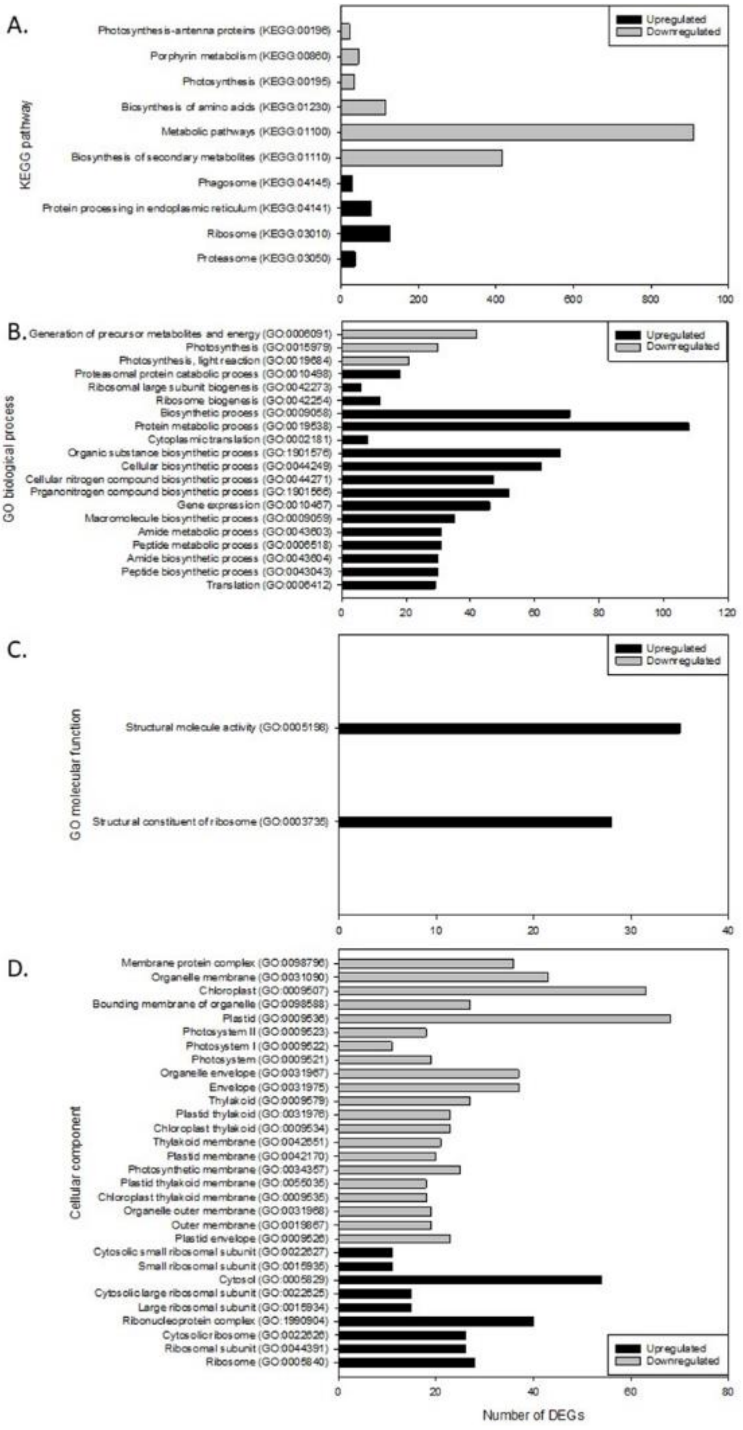

Figure S4: Enrichment analysis of DEGs for 1 h exposure to oxidative stress. A. KEGG pathway, B. GO biological process, C. GO molecular function, D. GO cellular component. The Y-axis shows the annotation terms and the X-axis the number of DEGs.

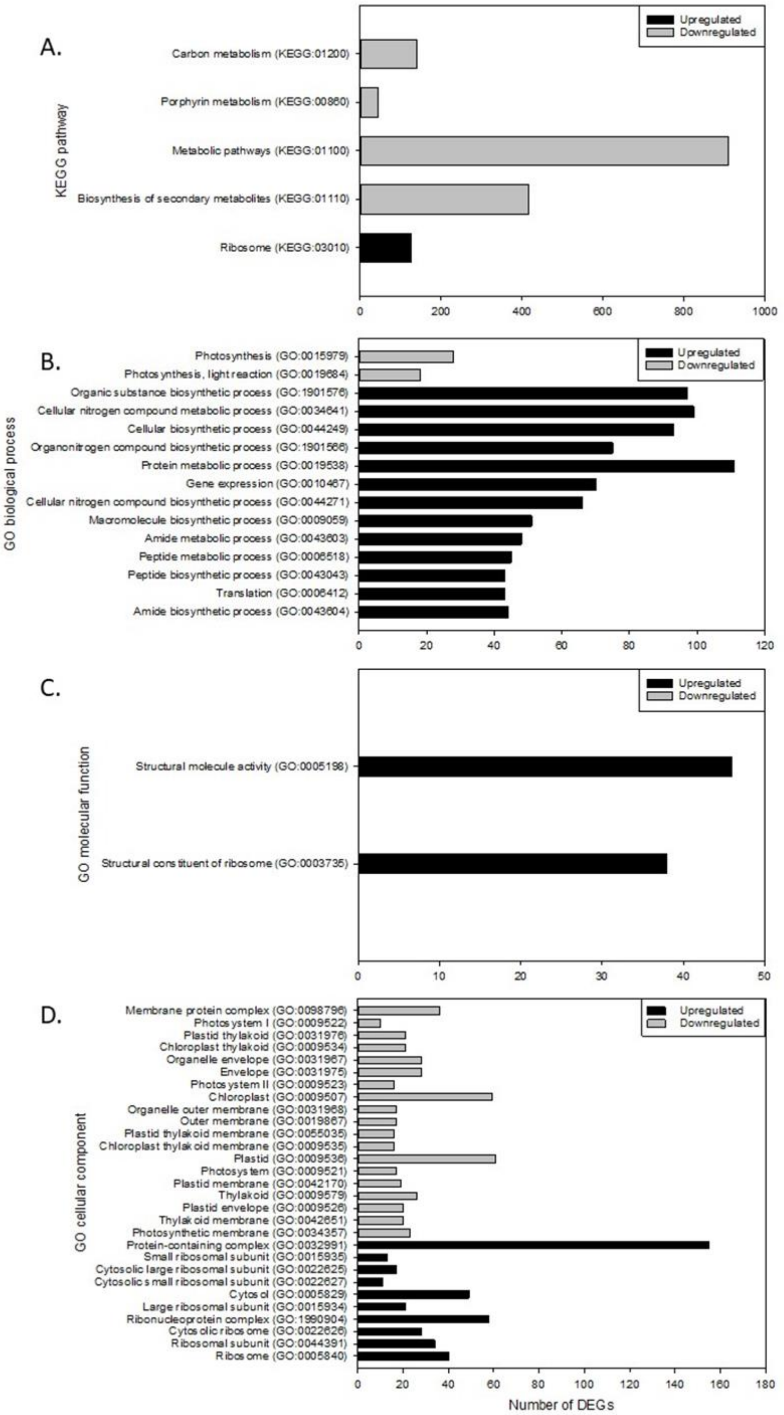

Figure S5: Enrichment analysis of DEGs for 6 h exposure to oxidative stress. A. KEGG pathway, B. GO biological process, C. GO molecular function, D. GO cellular component. The Y-axis shows the annotation terms and the X-axis the number of DEGs.

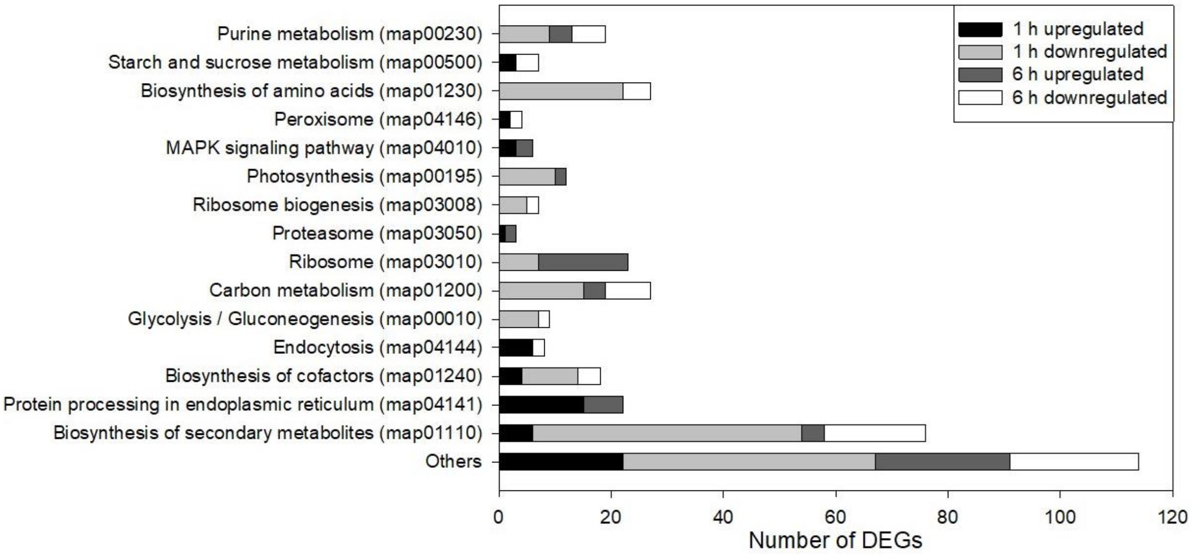

Figure S6. Number of DEGs per pathway being up-regulated and down-regulated in key metabolic pathways after 1 h and 6 h exposure to 0.5 mM H_2O_2.

Table S1: Primers used for RT-qPCR (data provided in excel file)

Table S2: RNAseq data for transcripts with adj.$p < 0.05$ (data provided in excel file)

Table S3: Relative response ratios of identified metabolites with GC/MS (data provided in excel file)
